# Chemokines as possible therapeutic targets in metastatic melanoma

**DOI:** 10.1002/cam4.6055

**Published:** 2023-05-11

**Authors:** Charlise Basson, June Cheptoo Serem, Priyesh Bipath, Yvette Nkondo Hlophe

**Affiliations:** ^1^ Department of Physiology, School of Medicine University of Pretoria Pretoria South Africa; ^2^ Department of Anatomy, School of Medicine University of Pretoria Pretoria South Africa

**Keywords:** chemokine, melanoma, metastasis, microenvironment, targeted therapy

## Abstract

**Background:**

Cutaneous melanoma is a relentless form of cancer which continues to rise in incidence. Currently, cutaneous melanoma is the leading cause of skin cancer‐related mortality, which can mainly be attributed to its metastatic potential. The activation of chemokine axes is a major contributor to melanoma metastasis through its involvement in promoting tumour cell migration, proliferation, survival, and adhesion. This review will focus on the role of chemokines in melanoma and possible therapeutic strategies to alter chemokine activation and subsequently inhibit the activation of signalling cascades that may promote metastasis.

**Methods:**

A literature review was conducted to evaluate chemokines as possible therapeutic targets in metastatic melanoma.

**Results:**

The crosstalk between signalling pathways and immune responses in the melanoma microenvironment resembles a complex and dynamic system. Therefore, the involvement of governing chemokine axes in the promotion of cutaneous and metastatic melanoma demands a proper understanding of the tumour microenvironment in order to identify possible targets and develop appropriate treatments against melanoma.

**Conclusion:**

Even though chemokine axes are regarded as promising therapeutic targets, it has become increasingly evident that chemokines can play a critical role in both tumour inhibition and promotion. The inhibition of chemokine axes to inhibit signalling cascades in target cells that regulate metastasis should, therefore, be carefully approached.

## Graphical abstract

Even though chemokine axes are regarded as promising therapeutic targets in melanoma, it has become increasingly evident that chemokines can play a critical role in both tumour inhibition and promotion. The inhibition of chemokine axes to inhibit signalling cascades in target cells that regulate metastasis should be carefully approached. This review will focus on the role of chemokines in melanoma and possible therapeutic strategies to alter chemokine activation and subsequently inhibit the activation of signalling cascades that may promote metastasis.

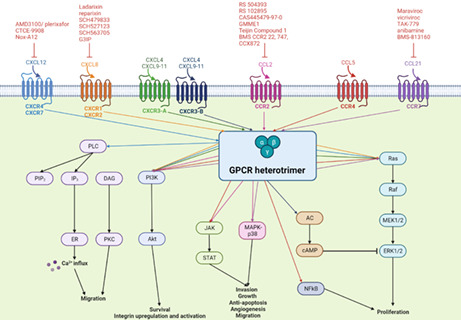

## 1 INTRODUCTION

Although melanoma is a rare type of cancer, accounting for less than 5% of all skin cancers, it is among the most lethal as it accounts for 80% of skin cancer mortality globally.^1^ Melanoma is a malignancy derived from melanocytes,^2^ which are pigment‐producing cells,^3^ derived from neural crest cells.^4^ These cells are prevalent in the epidermis, hair follicles, along mucosal surfaces, meninges and in the choroidal layer of the eye.^5^ This may result in the onset of melanoma in various organs, which can be classified as mucosal melanoma, ocular melanoma, or cutaneous melanoma. Cutaneous melanoma is the most prevalent type, occurring in over 90% of cases.^2^, ^6^ Cutaneous melanoma is a relentless form of cancer, with an incidence that continues to rise.^7^ Currently, cutaneous melanoma is the leading cause of death from skin cancer,^6^ which can mainly be attributed to its metastatic potential.^5^ As such, metastasis remains the primary cause of melanoma mortality, resulting in an estimated 5‐year overall survival rate of 23% in stage IV patients.^8^ Metastasis is the result of impaired regulation of several signalling pathways,^9^ which is often activated by the interaction between tumour‐associated chemokines and microenvironmental chemokine ligands.^10^

Current treatment strategies for melanoma include surgery, chemotherapy, targeted therapies, and immunotherapies.^11^ However, current melanoma therapies have various shortcomings, including adverse effects, lack of tumour cell specificity, and low efficiency as a result of drug resistance.^3^ To date, no therapeutic intervention has been successful against metastatic melanoma,^11^ and the continuous rise in mortality has highlighted the urgency for new treatment strategies.^12^ Chemokines, which are widely expressed in various cancers, including melanoma, represent promising targets to inhibit metastasis.^13^ Inhibiting chemokine‐mediated signalling pathways through chemokine antagonists represents a form of targeted treatment against melanoma that may induce adverse effects and greater clinical efficacy.^14^ Thus, identifying chemokines which are overexpressed in melanoma and understanding the axes associated with their activation may improve the management of metastatic melanoma burden by identifying selective inhibitors against chemokines. This review will focus on the role of chemokines in melanoma and possible therapeutic strategies to alter chemokine activation and subsequently inhibit the activation of signalling cascades that may promote metastatic parameters, such as proliferation, cell survival, migration, and adhesion.

## 2 CHEMOKINES AND THE TUMOUR MICROENVIRONMENT

Melanoma tumour cells exist in a tumour microenvironment,^15^ comprised of the extracellular matrix (ECM), connective tissue, and a variety of accessory cells such as stromal fibroblasts, endothelial cells, and immune cells.^16^ The tumour microenvironment provides structure and promotes tumour angiogenesis, proliferation, and survival.^16^, ^17^ Two primary components for signalling between malignant cells and the microenvironment include chemokine ligands and receptors.^18^ The mammalian chemokine network is comprised of over 50 chemokines^19^ and 18 chemokine receptors.^20^ Chemokines are representative of a superfamily of small peptide signalling cytokines^9^ with molecular weights of 8–10 kDa ^19^ and are secreted by stromal and endothelial cells.^19^ Chemokines play a role in physiological and pathological conditions, including processes such as organogenesis of the lymph node organs, inflammation, infection, tissue healing, and cancer.^9^ Structurally, chemokines can be divided into four groups (CXC, CC, XC, and CX_3_C) depending on the location of conserved cysteine residues,^18^, ^20^ where the C's are representative of N‐terminal cysteines, and the X's are representative of an amino acid.^21^ Chemokine receptors are activated upon binding to their cognate chemokine ligands.^22^ Chemokines usually bind exclusively in accordance with the group it represents (e.g. CXC chemokines bind to CXC receptors).^23^ However, many chemokines receptors can bind the same ligand and vice versa, which creates multiple combinations and results in multiple biological responses.^24^

## 3 THE ROLE OF CHEMOKINES IN MELANOMA

**FIGURE 1:**
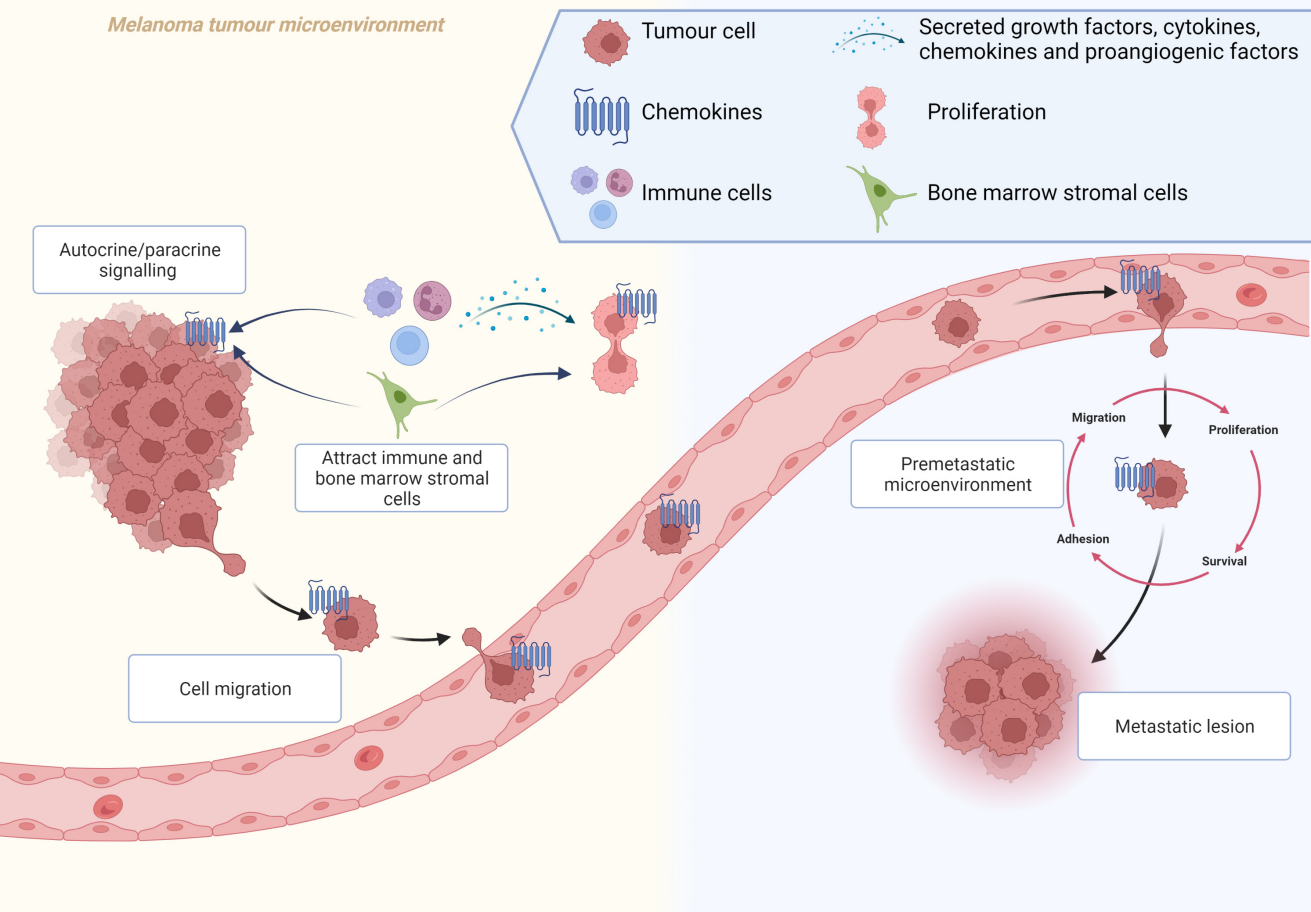
The intricate roles of chemokines in the melanoma tumour microenvironment. Chemokine receptors expressed by tumour cells can activate autocrine or paracrine signalling by binding to its cognate ligands. In addition, chemokines may also stimulate tumour cell proliferation as a result of 1) the recruitment of immune cells, which stimulate the secretion of growth factors, cytokines, chemokines, and pro‐angiogenic factors in the tumour microenvironment or 2) the recruitment of bone marrow stromal cells. Chemokines are also involved in cell migration, which is a key event in metastasis by allowing cancer cells to be retained in a premetastatic microenvironment that promotes tumour cell migration, proliferation, survival, and adhesion.^13^, ^14^, ^28^, ^29^, ^30^ Image created by Charlise Basson using BioRender (https://biorender.com/).

Metastatic melanoma cells are well‐known to express chemokine receptors which play major roles in metastasis, by creating migration gradients between cancer cells and organs, lymph nodes and lymphatic vessels which over‐express the corresponding ligand.^25^, ^26^ In vivo studies have demonstrated that the expression of the chemokine receptors CCR7, CCR10, CXCR3, and CXCR4 correlates with melanoma lymph node metastasis,^27^ which is regarded as an indication of tumour aggressiveness and is negatively associated with survival.^29^As such, it is generally assumed that chemokines predominantly favour tumour development in various ways (Figure 1).

First, many chemokines can function in an autocrine or paracrine manner, or they can attract immune cells, which can secrete growth factors, cytokines, chemokines, and pro‐angiogenic factors in the tumour microenvironment to induce cell proliferation.^13^ Second, chemokines are responsible for the migration of cells, which is essential for the recruitment of immune cells in inflammatory conditions, cell development, and metastasis.^13^, ^14^ The latter is achieved through the establishment of the directional gradient, which retains cancer cells in a premetastatic microenvironment that promotes tumour cell migration, proliferation, survival, and adhesion.^13^, ^28^ The tumour microenvironment contains a variety of cells, such as tumour cells, fibroblasts, immune cells, pericytes, endothelial cells, mesenchymal stem cells, adipocytes, red blood cells, and immune cells. Immune cells often divert from their expected anti‐tumour role, which suppresses cancer development. Instead, some immune cells that infiltrate cancer tissue may promote cancer progression by contributing to a pro‐tumorigenic microenvironment.^29^ Third, chemokines may direct and support the recruitment of bone marrow‐derived cells that suppress anti‐tumour immunity and promote tumour cell proliferation.^30^

## 4 THE CROSSTALK BETWEEN CHEMOKINES AND THE IMMUNE SYSTEM

Chemokines are crucial to direct immune cell migration to the tumour microenvironment in order to create an effective anti‐tumour immune response.^31^ However, research has indicated that immune cells in the tumour microenvironment may exert potent anti‐tumorigenic or pro‐tumorigenic responses and the balance between these responses are dependent on the stage of tumorigenesis, the state of immune cell activation, and the expression of chemokines on effector and regulatory target cells.^31^

Importantly, it is well‐known that tumour infiltration by T cells, especially Th1 and CD8+ T cells, is associated with favourable outcomes and improved patient survival.^32^ Anti‐tumour T cell responses are highly dependent on antigen‐presenting cells (APCs), especially type 1 dendritic cells (cDC1s) and cDC2s, that arrest and process tumour‐associated antigens (TAAs) for presentation to T cells.^33^ Chemokines recruit various immune cells, such as natural killer (NK) cells, cDC1 s, Th1 cells, and CD8+ T cells from the blood to the tumour microenvironment. NK cells, CD4+ T helper type 1 (Th1) cells and CD8+ T cells, can directly lyse cancer cells or produce cytokines to eradicate tumour cells.^31^, ^34^ As a result, NK cells are often associated with good prognosis in cancer patients.^35^ CD8+ T cells are often regarded as the most prominent anti‐tumour effector cells^34^ and are an integral part of adaptive and innate immune systems to differentiate between cancer and normal cells.^36^ Upon interaction with APC, CD8+ T cells have the ability to differentiate into cytotoxic T lymphocytes (CTLs), which destroy tumour cells in the tumour microenvironment by releasing perforin and granzyme B.^34^

In contrast, pro‐tumour effector cells include tumour‐associated macrophages (TAMs), myeloid‐derived suppressor cells (MDSC), tumour‐associated neutrophils (TANs) and regulatory T cells (Treg).^37^ TAMs are found in cutaneous melanoma and in several non‐melanoma skin cancers.^37^ TAMs are differentiated from monocytes and are known to display phenotypes of M2 (alternative‐activated macrophages, which are pro‐tumorigenic) as opposed to M1 (classical‐activated macrophages, which are anti‐tumorigenic).^38^ Upon the conversion of M1 to M2 phenotypes (known as macrophage polarisation), M2 phenotypes macrophages secrete anti‐inflammatory cytokines, including IL‐10, IL‐13 and IL‐4, along with pro‐angiogenic factors and metalloproteinases, which creates an immunosuppressive microenvironment conducive to tumour progression.^38^, ^39^ The expression of pro‐angiogenetic factors, including transforming growth factor β, vascular endothelial growth factor (VEGF), platelet‐derived growth factor and matrix metalloproteinases (MMPs) mediate angiogenesis^37^ by increasing the vascular leakiness, which attract pro‐tumorigenic chemokines to the site, such as CXCL8 (also referred to as IL‐8) and CXCL2, thereby promoting cancer cell invasion. Furthermore, in many skin cancers, including melanoma, TAMs may directly produce immunosuppressive chemokines,^31^ which may attract other immunosuppressive cells, including MDSCs, Tregs and TANs.^37^ MDSCs and TANs suppress T cells and NK cells and recruit Treg cells, which suppress T cell responses and promote cancer immune evasion and escape.^34^, ^35^

However, the crosstalk between signalling pathways and immune responses presents a complex and dynamic system in the context of cancer development and progression within the tumour microenvironment. Therefore, the involvement of governing chemokine axes in the promotion of cutaneous and metastatic melanoma demands a proper understanding of the tumour microenvironment in order to identify possible targets and develop appropriate treatments against melanoma. The major dysregulated chemokine axes, including the chemokine receptors corresponding ligands, types of cells on which they are expressed, pro‐ and anti‐tumour functions and inhibitors implicated in melanoma progression, are summarised in Table 1.

**TABLE 1:** Chemokine receptors in melanoma, corresponding ligands, types of cells on which they are expressed, pro‐ and anti‐tumour functions and possible inhibitors.

Chemokine receptors	Expressed on	Chemokine ligands	Expressed on	Protumour functions	Anti‐tumour functions	Inhibitors	References
CXCR1, CXCR2	Granulocytes, monocytes, mast cells, NK cells, primary tumour cells, neutrophils, basophils, CD8+ Teff cells, endothelial cells	CXCL8	Primary tumour cells	CXCR1: chemotaxis CXCR2: angiogenesis, invasion, migration	—	Ladarixin, reparixin, SCH479833, SCH527123, SCH563705, G3IP	[14, 31, 40]
CXCR3	Primary tumour cells, monocytes, T‐helper 1 (Th1) cells, CD8 T cells, natural killer T (NKT) cells, natural killer (NK) cells, dendritic cells (DC)	CXCL4, CXCL9‐11	CXCL9‐10: CD103 and DC, CXCL9‐11: tumour endothelial cells	Tumour cell migration, differentiation, activation of immune cells	Inhibition of cell proliferation, migration, apoptosis and angiogenesis	—	[30, 41, 42, 43]
CXCR4, CXCR7	Primary tumour cells, leukocytes and endothelial cells	CXCL12	Stromal cells, tumour cells, liver, lungs, bone marrow and lymph nodes	Cell migration, proliferation, survival, adhesion	—	AMD3100/ plerixafor, CTCE‐9908, Nox‐A12	[14, 19, 31, 41, 44, 45, 46]
CCR2	DC, endothelial cells, monocytes, macrophages, Th1 cells, basophils, NK cells, primary tumour cells	CCL2	Primary tumour cells, endothelial cells, fibroblasts, epithelial cells, smooth muscle cells myeloid cells	Melanoma invasion and growth, anti‐apoptosis, angiogenesis, and cell migration, recruitment of immune cells	—	RS 504393, RS 102895, CAS445479‐97‐0, GMME1, Teijin Compound 1, BMS CCR2 22, 747, CCX872	[47]
CCR4	Primary tumour cells, brain microenvironment, TH2, Treg cells, Th17 cells, CD8+ T cells, monocytes, B cells and immature DC	CCL17, CCL22	Primary tumour cells, brain microenvironment, astrocytes, microglia, endothelial brain cells	Melanoma metastasis to the brain	—	AF‐399/42018025	[31, 48, 49]
CCR5	T cells, macrophages, DC, eosinophils, microglia, monocytes, Th1 cells, NK cells, Treg cells, CD8+ T cells, neutrophils	CCL5, CCL3, CCL4 and CCL8	CCL5: T lymphocytes, macrophages, platelets, synovial fibroblasts, tubular epithelium, primary tumour cells	Tumour proliferation, survival, adhesion and migration, extracellular matrix remodelling, cancer stem cell expansion, treatment resistance, decreases in cytotoxicity of DNA‐damaging agents, deregulated metabolism, angiogenesis and immune cells	—	Maraviroc, vicriviroc, TAK‐779, anibamine, BMS‐813160	[31, 50, 51]
CCR7	Naïve, central memory and regulatory T cells, B cells, NKs, subsets of thymocytes and semi‐mature DC	CCL19, CCL21	Initial lymphatics	Lymph node metastasis	—	—	[27, 52, 53, 54]
CCR10	Keratinocytes of the epidermis, primary tumour cells	CCL27	Keratinocytes of the epidermis, primary tumour cells	Lymph node metastasis, tumour proliferation, invasion, and immune escape	—	—	[54, 55, 56, 57]

Abbreviations: CTLs, cytotoxic T lymphocytes; DC, dendritic cells; DNA, deoxyribonucleic acid; NK, natural killer; TAMs, tumour‐associated macrophages; myeloid‐derived suppressor cells, MDSC; TANs, tumour‐associated neutrophils; Th, T helper; Treg, regulatory T cells.

## 5 THE CXCR4/CXCR7/CXCL12 AXIS

The role of the chemokine receptor, CXCR4 is highlighted in cancer, due to its ubiquitous expression by various cancers,^23^, ^58^ including melanoma.^59^ CXCR4 is a G‐protein‐coupled receptor (GPCR).^60^ In addition to its physiological expression on leukocytes and endothelial cells,^31^ tumour‐associated expression of CXCR4 is a well‐known independent prognostic marker for several cancers^44^ and is associated with elevated mortality rates in melanoma patients.^61^, ^62^ A previous study by Scala et al. performed membrane and cytoplasmic staining for CXCR4 expression in primary cutaneous melanoma patients and found that CXCR4 expression correlated with poor prognosis and survival.^63^ In addition, pre‐clinical evidence using B16 F10 melanoma cell cultures demonstrated that CXCR4 promotes melanoma growth^64^ as well as the formation and progression of pulmonary metastatic nodules.^65^ Other previous studies have indicated that the over‐expression of CXCR4 is associated with lymph node metastasis, ulceration, tumour thickness,^62^ the stage the tumour is in and overall survival.^62^

**FIGURE 2:**
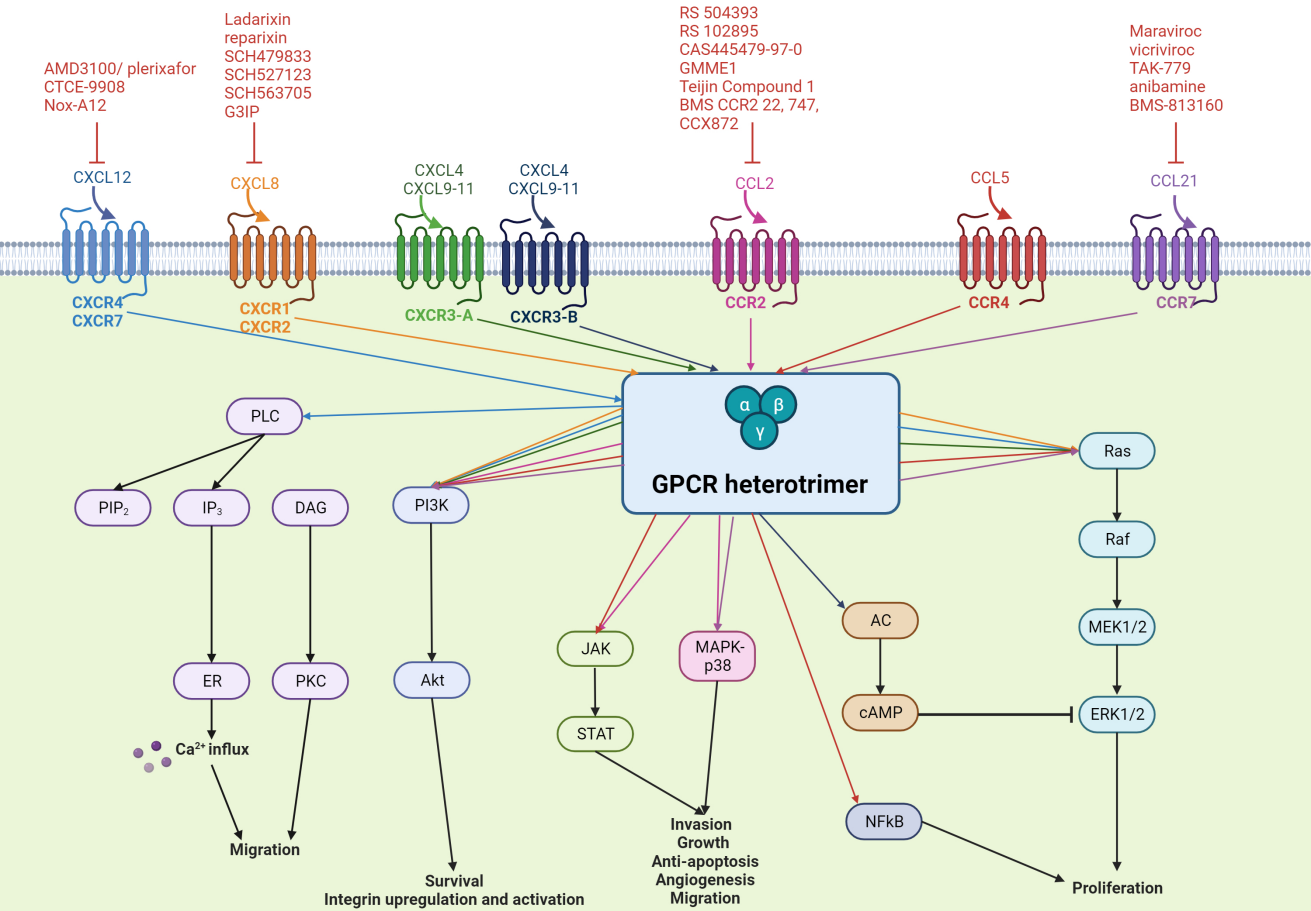
A schematic diagram of major activated chemokine‐mediated signalling pathways and possible inhibitors in melanoma. Chemokines activate the appropriate GPCR heterotrimeric subunit (Gα, Gβ and Gγ), which upon activation facilitates the dissociation of the subunit and favours the activation of various signalling cascades. Major activated signalling pathways include MAPK, PI3K/AKT, phospholipase C, MAPK‐p38, adenyl cyclase and JAK/STAT. AC, adenyl cyclase; AKT, protein kinase B; ATP, adenosine triphosphate; Ca^2+^, calcium ions; cAMP, cyclic adenosine monophosphate; CXCL12, CXC chemokine ligand 12; CXCR4, CXC chemokine receptor 4; DAG, diacylglycerol; ER, endoplasmic reticulum; ERK, extracellular signal‐regulated kinase; GDP, guanosine diphosphate; GPCR, G‐protein‐coupled receptor; GRK, G‐protein‐coupled receptor kinase; GTP, guanosine triphosphate; IP3, inositol‐1,4,5‐trisphosphate; JAK, Janus kinas; MAP‐K, mitogen‐activated protein kinase; MEK1/2, MAPK/ERK kinase1/2; NFkB, nuclear factor kappa B; PI3K, phosphoinositide 3‐kinase; PIP_2_, phosphatidylinositol bisphosphate; PKC, protein kinase C; PLC, phospholipase C; Raf, rapidly accelerated fibrosarcoma; Ras, rat sarcoma virus; STAT, signal transducer and activator of transcription.^14^, ^18^, ^40^, ^41^, ^46^, ^47^, ^50^, ^69^, ^71^, ^72^, ^73^, ^77^, ^85^, ^100^, ^107^, ^108^ Image created by Charlise Basson using BioRender (https://biorender.com/).

The corresponding ligand, CXCL12 was previously known for its role in lymphocyte trafficking to the bone marrow to regulate haematopoiesis.^66^ More recently, tumour cell migration and metastasis as functions of CXCL12, have been confirmed.^67^ As a result, tumour‐associated CXCL12 expression attracts CXCR4‐expressing inflammatory, vascular and stromal cells to the tumour microenvironment,^45^ such as the bone marrow^68^ where tumour growth, survival, invasion and metastasis is supported.^14^ Autocrine activation, occurring as a result of the interaction between CXCR4 and CXCL12 expressed by tumour cells, promotes proliferation and invasion, whereas paracrine signalling, occurring due to the interaction between CXCR4 on tumour cells and CXCL12 on microenvironmental stromal cells, promotes tumour proliferation and metastasis to secondary sites.^13^ These cellular processes are achieved through the CXCR4/CXCL12 axis, which activates several downstream molecular signalling pathways, including the phospholipase C (PLC) pathway to promote cell migration and the release of intracellular calcium; the mitogen‐activated protein kinase (MAP‐K) pathway to primarily promote proliferation; the phosphoinositide 3‐kinase/ protein kinase B (PI3K/AKT) pathway to increase cell survival^18^ and the upregulation of integrin expression and activation on tumour cells to enhance adhesion (Figure 2).^69^ A previous study by Cardones et al., demonstrated the importance of the adhesion molecules, namely beta1 integrins in melanoma metastasis with in vitro and in vivo experiments. This study found that CXCR4‐expressing B16 cells demonstrated enhanced binding affinities when exposed to CXCL12. In addition, metastasis of CXCR4‐expressing B16 cells to the lungs was strongly inhibited by anti‐CXCL12.^70^

A known CXCR4 inhibitor, CTCE‐9908 is derived from human CXCL12^71^, ^72^ and has been shown to reduce metastasis in eight different murine cancer models through CXCR4 inhibition.^71^ The Food and Drug Administration (FDA) granted CTCE‐9908 an orphan drug status (given to drugs that show promise to treat rare diseases) for the treatment of osteogenic sarcoma.^73^ This 17‐amino acid peptide^19^ is a peptide analogue of CXCL12^74^ and contains an altered NH_2_‐terminal sequence,^71^ which hinders the binding between the ligand and the receptor^74^ by competitively binding to CXCR4.^72^ By disrupting receptor phosphorylation, CTCE‐9908 previously inhibited cellular responses associated with downstream signalling pathways of the CXCL12/CXCR4 axis and ultimately resulted in decreased levels of adhesion, proliferation and migration in several cancers.^19^ A previous study by Kim et al. demonstrated that mice, injected with CXCR4‐B16 cells and treated with CTCE‐9908 had a 50% decrease in the number of metastatic lung nodules. Importantly, this study also showed that the administration of CTCE‐9908 prior to the injection of CXCR4‐B16 cells into the mice, resulted in an 80% decrease in the number of metastatic lung nodules. This study hypothesised that CXCR4 inhibition, prior to metastasis, may result in an improved outcome as the CXCR4 inhibitor may interfere with the ligand more effectively.^61^ In addition, Hassan et al., previously indicated that CTCE‐9908 also inhibited vascular endothelial growth factor in a transgenic mouse model. This is of particular importance, as VEGF (a metastatic promoting molecule) is known to upregulate CXCR4 expression, which in turn promotes VEGF expression.^75^

Other inhibitors of the CXCR4/CXCL12 axis, such as AMD3100 or plerixafor,^46^ Nox‐A12, a pegylated structured L‐oligoribonucleotide that binds and neutralises CXCL12,^76^ has previously demonstrated cytotoxicity in pre‐clinical in vivo studies.^41^ In addition, AMD3100 previously inhibited proliferation and migration in uveal melanoma cell lines.^77^ Interestingly, AMD3100 is not orally bioavailable and must be injected subcutaneously.^78^ This led to the development of AMD11070, which is a novel orally bioavailable CXCR4 inhibitor with an improved pharmacokinetic profile. AMD11070 previously inhibited CXCR4/CXCL12 interactions in a CXCR4‐expressing T cell line, while showing no effect towards other chemokine receptors, such as CXCR3, CXCR7, CCR1, CCR2b, CCR4, CCR5 or CCR7.^79^

However, the efficacy of these inhibitors has recently been challenged^41^ after the discovery that chemokines often display fluctuant behaviour by allowing various types of chemokine receptors to bind with it.^23^ It has been found that CXCR7 is another receptor for CXCL11 and CXCL12.^80^ As a result, CXCR7 can also interact with CXCL12 to activate downstream signalling pathways associated with this axis.^27^ Although CXCR7 is not a GPCR like CXCR4,^80^ CXCR7 activate various signalling modules, such as ERK, AKT/mTOR and Src kinase.^81^ In addition, a recent study has demonstrated that elevated CXCR7 expression correlates with worse overall survival in melanoma patients.^81^ The chemokine CXCR7 is well‐known for its ability to act as a ‘decoy’ receptor, as the internalisation of CXCL11 or CXCL12 bound to CXCR7 generates a chemokine gradient, even in the absence of signalling.^41^

As proof of concept, a previous in vitro study, using antibodies or siRNA previously induced CXCR7 inhibition in prostate cancer cell lines, which altered cell proliferation. Similarly in vivo evidence of siRNA‐mediated CXCR7 inhibition showed delayed tumour growth and inhibition of invasion in endometrial carcinoma and oesophageal cancer mice models.^82^ Two CXCR7 inhibitors (CCX771 and CCX662) were previously investigated in an in vivo study. In combination with these two CXCR7 inhibitors, irradiation inhibited tumour recurrence in three rodent glioblastoma multiforme models (mouse U251 model, rat ENU model and rat C6 model). The effects of a small‐molecule CXCR7 antagonist, namely ACT‐1004‐1239, has recently been investigated in a human cohort, consisting of 30 healthy male and female subjects between 18 and 55 years. This study found that ACT‐1004‐1239 presented with a favourable safety/tolerability and pharmacokinetic profile.^83^ However, the effects of these inhibitors against melanoma cell lines, animal models and human cohorts are yet to be determined.

## 6 THE CXCR3/CXCL9,10,11 AXIS

CXCR3 (GPR9/CD183) is an interferon‐inducible chemokine receptor, which is predominantly expressed on monocytes, Th1 cells, CD8 T cells, NKT cells, NK cells, DC and cancer cells.^30^ An in vitro study previously indicated that CXCR3 is expressed in a highly metastatic melanoma cell line (B16 F10) as well as a low metastatic melanoma cell line (B16 F1). In the same study, these findings were substantiated with immunohistochemical staining of primary melanoma tissue samples, which showed that five out of nine patients expressed CXCR3.^84^ The expression of CXCR3 is upregulated under inflammatory conditions due to cytokine stimulation,^30^ such as in patients with melanoma^41^ and attracts immune cells to sites of interferon‐mediated inflammation.^30^ According to Bedognetti et al. CXCR3 chemokine ligands, including CXCL9, CXCL10 and CXCL11, are highly expressed in metastatic melanoma patients.^85^ Interferon gamma (IFN‐γ) induces CXCL9, while IFN‐γ and type I interferons induces CXCL10 and CXCL11.^30^ An in vitro study previously demonstrated that B16 F10 cells undergo directional migration and invasion in response to CXCL9, CXCL10 and CXCL11. In the same study, an in vivo investigation where C57BL/6 mice injected with B16 F10 cells confirmed that CXCR3 signalling in B16 F10 cells correlate with its metastasis to the lungs and the lymph nodes, where CXCL9 and CXCL10 is expressed.^84^

The complexity of chemokine signalling can further be increased by introducing the idea that the same ligand may exert different functions based on the receptor it binds to, for example, CXCR3. The paradoxical effects of CXCR3 can mainly be attributed to the fact that various isoforms of CXCR3, namely CXCR3‐A, CXCR3‐B and CXCR3‐alt, may demonstrate opposing functional abilities.^86^ The dual functionality of CXCR3 receptors represents a double‐edged sword, as its immune‐associated expression can promote anti‐tumour immune responses through paracrine signalling that promotes immune activation, while its tumour‐associated expression promotes cancer cell proliferation and metastasis through autocrine signalling.^30^, ^41^, ^42^, ^43^

Paracrine secretion of CXCR3 results in the migration, differentiation, and activation of immune cells through the recruitment of cytotoxic lymphocytes, natural killer cells, and macrophages. First, activating toll‐like receptors and ribonucleic acid helicases from melanoma cells stimulates the secretion of IFN‐a/β from histiocytes and endothelial cells, which secrete CXCL10. The chemokine CXCL10 recruits NK and CD4+ Th1 cells from the blood into the target tissue. The NK and CD4+ Th1 cells then produce IFN‐γ, which leads to the secretion of CXCL9, CXCL10 and CXCL11 by dendritic and other resident cells. The secreted CXCL9, CXCL10 and CXCL11 interact with tumour cells to induce cell death, such as apoptosis and attract adaptive immune cells leading to anti‐tumour immunity.^30^, ^41^

In terms of autocrine signalling, metastatic effects and cell proliferation are promoted by the interaction between CXCL11 and CXCR3‐A (expressed in epithelial cells),^41^, ^85^ via PI3K and ERK1/2 pathways through Gi activation (Figure 2).^41^ As a result, CXCR3 correlates with poor patient survival and metastasis through the activation of cell processes that promote tumour cell proliferation, survival and migration.^30^, ^41^ In addition, the interaction of CXCL11 with CXCR3‐B (expressed in fibroblasts, endothelial and epithelial cells) activates Gαs, which stimulates adenyl cyclase to increase cyclic adenosine monophosphate (cAMP) concentrations, which inhibits ERK1/2 and results in the inhibition of metastatic parameters, such as cell proliferation, migration and apoptosis^41^ and angiogenesis (Figure 2).^85^

A previous in vitro study showed moderate CXCR3 expression in a human melanoma cell line (BLM), lower expression in amelanotic cutaneous melanoma cell line (SK‐Mel 37) and low expression in human malignant melanoma cell lines, such as MeWo and A375 cells. This study also demonstrates that CXCL9 significantly increased phosphorylation of p38 MAPK in the BLM cell line, (which showed the highest CXCR3 expression) and no phosphorylation of p38 MAP kinase was observed in the MeWo cell line (which showed the lowest CXCR3 expression). This study also found that the CXCR3/CXCL9 axis enhances melanoma cell adhesion and may therefore also promote melanoma cell proliferation, survival and apoptosis.^87^ Furthermore, the CXCR3‐B/CXCL11 axis may inhibit vascular endothelial growth factor inhibitor (VEGF)/VEGFR2 and downstream modules, including phospholipase Cγ, p‐JNK and p‐ERK.^85^

Another isoform of CXCR3, namely CXCR3‐alt is co‐expressed with CXCR3‐A and leads to cell migration upon its activation by CXCL11. Ligands namely CXCL9, CXCL10, CXCL11 have demonstrated to activate both the isoforms, whereas CXCL4 selectively activates CXCR3‐B.^41^

Synergistic effects against established subcutaneous melanoma tumours were previously observed with treatment involving intra‐tumoural injection of CXCL10 or CXCL9 combined with IL‐12 treatment and was reported to cure 80% of mice. It is hypothesised that the synergism can mainly be attributed to T cell recruitment by CXCL10 or CXCL9 expression followed by IL‐12 or IL‐2‐mediated activation.^24^ A phase I/II clinical trial, investigating tebentafusp (a bispecific fusion protein targeting gp100) in metastatic melanoma patients reported an increase in serum CXCL10 and reduction in circulating CXCR3+ CD8+ T cells. Overall, tebentafusp resulted in a 65% 1‐year survival rate in metastatic uveal melanoma and metastatic cutaneous melanoma patients.^88^

## 7 THE CXCR1,2/CXCL8 AXIS

The chemokine receptors CXCR1 and CXCR2 are expressed on granulocytes, monocytes, mast cells, NK cells, neutrophils, basophils, CD8+ Teff cells and endothelial cells,^31^ whereas being overexpressed in melanomas.^14^, ^40^ Pre‐clinical evidence suggests that tumour‐associated CXCR1 and CXCR2 overexpression correlates with increased proliferation and microvessel density and reduced apoptosis in melanoma.^14^ It has previously been demonstrated that CXCR1 activation mediates chemotaxis, whereas CXCR2 activation promotes angiogenesis, invasion and migration of human melanoma cells.^40^ Cellular responses, such as tumour cell survival and proliferation, are the consequences of CXCR1 and CXCR2 mediated activation of (PI3K)/Akt and MAPK signalling pathways.^14^ In addition, it has previously been reported that this axis promotes chemotherapy resistance in melanoma, as dacarbazine chemotherapy‐induced upregulation of CXCR2 by a nuclear factor kappa B (NF‐κB)‐dependent mechanism.^89^

The corresponding ligand to CXCR1 and CXCR2, namely CXCL8, is overexpressed on various cancer cells, including melanoma.^40^ The overexpression of CXCL8 in melanoma is associated with disease progression,^40^ as an increased expression of CXCL8 and CXCR1 and CXCR2 has previously been associated with melanoma transition from a radial (early stage) to vertical (later stage) growth phase.^14^ In addition, CXCL8 expression in melanoma has been associated with angiogenesis and metastasis in pre‐clinical animal models by promoting vascularisation, MMP‐2 activation and anoikis resistance.^40^

In addition to the effect of the CXCR1,2/CXCL8 axis in melanoma cells, it also plays an essential role in immune regulation as CXCR1 and CXCR2 are expressed on neutrophils, correlating with poorer prognosis and poor response to checkpoint inhibition.^14^ The involvement of the immune system also creates a positive feedback loop, as the neutrophils in the tumour microenvironment also produce CXCL8, which leads to further neutrophil recruitment.^14^

A variety of CXCR1/2 antagonists have been developed over the decades.^40^, ^90^ However, the CXCR1/2 antagonist tested against melanoma includes Ladarixin, SCH527123, SCH479833 and ABX‐IL8.^40^, ^90^ SCH‐527123 previously inhibited melanoma cell proliferation, chemotaxis and invasion through the inhibition of the PI3K/AKT pathway and induced apoptosis.^91^ In addition, SCH479833 inhibited tumour cell proliferation and micro vessel density in a melanoma mouse model.^90^ An in vivo study, on mice bearing human melanoma cells (A375SM and TXM‐13), demonstrated that ABX‐IL8 significantly reduced tumour growth, supressed experimental metastasis and angiogenesis and induced apoptosis.^92^ Ladarixin, a dual CXCR1/2 inhibitor, previously inhibited motility and induced apoptosis in cutaneous and uveal melanoma cells through the inhibition of AKT and NF‐kB signalling pathways.^93^ In addition, the inhibition of the CXCR1,2/CXCL8 axis in mouse models reduced melanoma infiltration by neutrophils, tumour growth, angiogenesis and metastasis.^14^

## 8 THE CCR2/CCL2 AXIS

The CCR2/CCL2 axis is a known contributor to tumour cell survival and invasion, angiogenesis, and the recruitment of immune cells.^47^ Various cell types, including DC, endothelial cells, monocytes, macrophages, Th1 cells, basophils, NK cells and various cancer cells express CCR2, which binds to CCL2. The aforementioned ligand is produced by tumour cells, endothelial cells, fibroblasts, epithelial cells, smooth muscle cells and myeloid cells.^47^ Elevated CCL2 levels have been reported in melanoma patients, which was associated with tumour metastasis, immunosuppression^94^ and disease progression as it mediates the invasion and growth of metastatic melanoma.^54^ The involvement of the CCR2/CCL2 axis is of particular importance in the early stages of tumour invasion^14^ and is regarded as a predictor of poor prognosis in cancer patients.^47^

Cellular processes, such as invasion and growth of metastatic melanoma along with anti‐apoptosis, angiogenesis, and cell migration are promoted by the CCR2/CCL2‐mediated activation of phosphatidylinositol 3‐kinase (PI3K)/AKT, mitogen‐activated protein kinase (MAPK)/p38 and Janus kinase (JAK)/STAT3 signalling pathways (Figure 2).^47^ In addition, a previous study supported this by demonstrating that CCL2‐neutralising antibodies inhibited tumour growth in mouse models by suppressing CCL2 gene expression.^95^

In addition to its direct effects on melanoma cell development and progression, CCL2 also plays a vital role in the recruitment of immune cells, such as MDSCs and monocytes, which differentiate into TAMs to initiate a microenvironment conducive to tumour cell proliferation.^47^ Much progress has been made in terms of the development of inhibitors against this axis. Inhibitors of this axis previously demonstrated pre‐clinical evidence of anti‐cancer effects through antagonising CCR2.^47^ Of these inhibitors, only a few have progressed to clinical trials in various cancers.^47^ Although the effects of these inhibitors have not been tested on melanoma patients, clinical trials have reported important observations regarding the safety and efficacy of these inhibitors in humans.

Phase 1 clinical trials (NCT00537368 and NCT01204996) and a phase 2 clinical trial (NCT00992186) investigating a mAb to human CCL2 (CNTO888, carlumab) demonstrated an increased affinity of CNTO888 for CCL2 in subjects (dissociation constant of 2.4 nmol/L), which suggest a reduced ability of carlumab to inhibit CCL2.^96^ Furthermore, it has been reported that carlumab has been discontinued due to toxicity in subjects that did not resolve within 2 weeks.^97^ Another phase 2 clinical trial (NCT01015560) investigating a mAb to human CCL2 (MLN1202, plozalizumab) in bone metastasis of unspecified tumours was conducted in a cohort of 44 subjects.^47^, ^96^ However, 7% of these subjects experienced serious adverse effects.^96^

In addition, clinical trials were conducted on small‐molecule antagonists of CCR2. One of these inhibitors, namely PF‐04136309 was investigated during a phase 1 clinical trial (NCT01413022) in patients with borderline resectable and locally advanced pancreatic cancer. It was reported that PF‐04136309 prevented bone marrow‐derived CCR2+ monocyte mobilisation into the peripheral circulation, which resulted in a decrease in the amount of TAMs.

## 9 THE CCR4/CCL17,22 AXIS

The CCR4/CCL17,22 axis has revealed its particular importance in melanoma brain metastasis,^98^ which is predominantly mediated by the interaction between CCR4 and its ligands, namely CCL17 and CCL22. The receptor and the ligands of this axis are expressed on metastatic melanoma cells, in the brain microenvironment^48^ and immune cells, such as TH2, Treg,^49^ Th17 cells, CD8+ T cells, monocytes, B cells and immature DC.^31^ Its ligands are commonly expressed by astrocytes, microglia and endothelial brain cells.^48^ In vitro and in vivo studies have confirmed the association between CCR4 overexpression and increased primary tumour growth and brain metastasis.^54^, ^99^

Interestingly, the metastatic migration of melanoma cells to the brain is solely mediated by astrocyte‐associated CCR17 secretion, which plays a crucial role in the establishment of a premetastatic microenvironment in the brain.^48^ The conclusion that the CCR4/CCL17,22 axis promotes melanoma metastasis to the brain was previously substantiated by a variety of findings, including the fact that: (1) CCR4 expression is upregulated in melanoma metastasis, which elevates its tumorigenicity and potential to form brain metastasis; (2) CCR4 upregulation elevated that malignancy phenotype of melanoma cell populations and (3) a CCR4 antagonist (AF‐399/42018025) in melanoma‐bearing mice inhibited local tumorigenicity and metastasis formation.^98^

## 10 THE CCR5/CCL5 AXIS

Under physiological conditions, CCR5 is expressed on various cell types, including T cells, macrophages, DC, eosinophils, microglia, monocytes, Th1 cells, NK cells, Treg cells, CD8+ T cells and neutrophils,^31^ but is overexpressed in various cancers, such as melanoma.^50^ As such, it correlates positively with tumour progression and metastasis.^50^ The CCR5/CCL5 axis was previously described as detrimental and is known to always result in the enhancement of tumour cell survival.^100^ The ligand CCL5 can bind to CCR1, CCR3, and CCR4.^100^ Even though CCR5 can be activated by various ligands, including CCL5, CCL3, CCL4 and CCL8^100^ it has been established that CCL5 displays the highest affinity to CCR5.^100^ The ligand, CCL5 is expressed in T lymphocytes, macrophages, platelets, synovial fibroblasts, tubular epithelium and various cancers, such as melanoma.^51^

The activation of CCR5 leads to the activation of signalling pathways, including PI3K/Akt, JAK/STAT3, HIF‐α, MAPK/ERK and NF‐kB.^100^ The activation of these pathways promote cytoskeleton rearrangement and results in increased tumour proliferation, extracellular matrix remodelling, tumour cell migration, cancer stem cell expansion, treatment resistance, decreases the cytotoxicity of DNA‐damaging agents, deregulated metabolism, angiogenesis and immune, stromal cell recruitment^50^ and the activation of αvβ3 integrins.^51^ Integrin activation in melanoma has been shown to favour cell adhesion and invasion in vitro, and experimental metastasis in vivo.^13^

In terms of its immunomodulatory function, the CCL5/CCR5 axis is known for creating a hospitable microenvironment for tumour cells by recruiting immunosuppressive cells, such as Tregs, MDSCs and TAMs to the tumour.^100^ This was supported by a previous study where CCR5‐deficient mice demonstrated delayed melanoma growth due to decreased recruitment of Tregs.^51^ A study by Blattner et al. demonstrated that the CCR5/CCR5 axis contributes to MDSC recruitment and activates the immunosuppressive functions of MDSC in the tumour microenvironment.^101^

A previous study, investigating CCR5 knockout (CCR5−/−) and wild‐type (CCR5+/+) mice, reiterated the important role of CCR5 in melanoma progression, as apoptosis, inhibition of NF‐κB and upregulation of IL‐1Rα was observed in CCR5‐deficient mice.^51^ In vivo, TAK‐779 previously inhibited cell proliferation, metastasis and Tregs migration into tumours in a in a murine model of pancreatic cancer.^102^

Due to the overexpression of CCR5 in several cancers, pyrimidine small‐molecule CCR5 inhibitors, namely maraviroc and vicriviroc, and a monoclonal anti‐CCR5 antibody, namely leronlimab, which were primarily used against human immunodeficiency virus (HIV), have been repurposed in clinical trials as cancer treatment.^103^ Three independent studies previously demonstrated that CCR5 inhibitors, including maraviroc, vicriviroc and leronlimab inhibited breast cancer metastasis and promoted cell death of DNA‐damaging chemotherapies.^103^ All three of these inhibitors are currently being investigated in clinical trials.^103^

## 11 THE CCR7/CCL21 AXIS

CCR7 is expressed on immune cells, including naïve, central memory and Tregs, B cells, NKs, subsets of thymocytes and semi‐mature DC that migrate to and within lymphoid organs.^52^ DC‐ associated CCR7 expression previously enhanced CD8 T‐cell‐driven antitumor immune responses, which are associated with increased survival rates.^104^ However, CCR7 is upregulated in cancer, such as melanoma, and its expression seems to be linked to cancer aggressiveness, as CCR7 expression is higher in metastatic melanoma cells than in melanoma cells at the primary tumour site.^52^ Mounting evidence suggests that tumour‐associated CCR7 expression mediates migration towards CCL21‐expressing lymphoid organs.^105^ This mechanism may explain the association between melanoma and lymph node metastasis,^27^ potentially pointing towards an escape mechanism of CCR7 expressing T cells and DC to avoid tumour immune infiltration.^54^, ^99^

Melanoma‐associated CCR7 upregulation previously correlated with decreased survival^106^ and resulted in metastasis formation in draining lymph nodes.^107^ Decreased survival associated with the activation of CCR7 is achieved through PI3K/Akt, ERK1/2, JNK and p38 signalling (Figure 2).^107^, ^108^ In addition, a previous study, investigating the expression of CCL21 by B16 F10 melanoma cells documented the elevated infiltration of TREG cells and myeloid‐derived suppressor cells into tumours and found that CCL21 may be a driver of lymphoid neogenesis and immunosuppression, resulting in melanoma cell proliferation.^109^

Despite the correlation of the CCR7/CCL21 axis with melanoma metastasis to the lymph nodes, there are currently no inhibitors on the market to inhibit the downstream effects of this axis.^53^ However, a CCL21‐specific antibody has previously blocked metastasis, which confirms the importance of the CCR7/CCL21 axis in metastasis.^54^

## 12 THE CCR10/CCL27 AXIS

Both CCR10 and its corresponding ligand, CCL27, is expressed in normal and inflammatory skin,^55^, ^56^ specifically by keratinocytes of the epidermis.^57^ Under physiological conditions, CCL27 is known to recruit CCR10‐expressing leukocyte antigen CLA+ T cells to the skin when inflammation occurs.^55^ As such, the CCR10/CCL27 axis promotes directional migration of immune cells to the skin.^99^ Overall, moderate expression of the ligand, CCL27 correlates with T lymphocyte density. However, surprisingly, high CCR10 expression is associated with lower T cell density.^99^ A previous study suggests that some human keratinocyte‐derived skin tumours may downregulate CCL27 expression to prevent T cell attraction and subsequent antitumor immunity.^110^ However, another study hypothesised that T cells expressing CCR10 are unable to infiltrate melanoma lesions expressing CCL27. This might explain why high CCR10 expression is associated with poor survival, thick primary lesions and is negatively associated with intratumoral T cell density.^99^ However, consensus on this topic has not been reached and further in‐depth research is needed to investigate the role of the CCR10/CCL27 axis on immune cells.

The CCR10/CCL27 axis plays a significant role in tumour development, which is illustrated by the link between CCR10 overexpression in primary melanoma cells and increased regional lymph node metastasis.^54^ The correlation between CCR10 and lymph node metastasis was demonstrated in B16 F1 cells, where CCR10 overexpression induced an immunosuppressive environment, leading to tumour growth and lymph node metastasis.^99^ In addition to promoting melanoma metastasis to the lymph nodes,^111^ the CCR10/CCL27 axis is also involved in mediating tumour proliferation, invasion, and immune escape, thus directly correlating with cancer progression.^55^ Currently, there are no inhibitors actively being tested against cancer.

## 13 FUTURE DIRECTIONS AND CONCLUSIONS

Chemokines are key molecules involved in many physiological processes, such as cell migration, proliferation, survival and differentiation. However, chemokines have also proven their involvement in pathological conditions, such as cancer. In particular, melanoma is known to overexpress many chemokines that aid tumour initiation, progression and metastasis through interaction with the microenvironment. As reviewed in this manuscript, metastasis as a result of microenvironmental chemokine receptor‐ligand interactions, is predominantly achieved through the activation of downstream signalling pathways, which promote metastatic parameters while retaining tumour cells in a premetastatic environment and recruiting immune cells. The major chemokine/chemokine receptor interactions occurring in melanoma development and progression include the CXCR4/CXCR7/CXCL12, CXCR3/CXCL9,10,11, CXCR1,2/CXCL8, CCR2/CCL2, CCR4/CCL17,22, CCR5/CCL5, CCR7/CCL21 and CCR10/CCL27 axes. As such, chemokines represent attractive potential therapeutic targets to manage the melanoma burden.

However, as our knowledge of chemokines and their axes has improved over the past few decades, it has become increasingly evident that chemokines can play a critical role in both tumour inhibition and promotion. The inhibition of chemokine axes to inhibit signalling cascades in target cells that regulate metastatic parameters such as proliferation, cell survival, migration, and adhesion should, therefore, be carefully approached. This may explain the presence of limited approved inhibitors to target chemokines in cancer, specifically melanoma. As such, a deep understanding of the entire tumour microenvironment is crucial to understand the dynamic interplay in the melanoma chemokine network. However, the association between chemokine overexpression and melanoma progression and metastasis warrants further investigation to (1) identify and develop possible inhibitors against hyperactivated chemokine axes in melanoma and (2) critically evaluate its beneficial and disadvantageous effects in pre‐clinical and clinical studies.

In conclusion, after evaluating chemokine axes and their inhibitors on melanoma patients, the development of combined approaches to target chemokine axes along with other aberrant signalling pathways in melanoma might represent a promising strategy to manage the melanoma burden and improve clinical outcomes.

## AUTHOR CONTRIBUTIONS

**Charlise Basson:** Conceptualization (equal); investigation (equal); writing – original draft (lead). **June Cheptoo Serem:** Conceptualization (equal); supervision (equal); writing – review and editing (equal). **Priyesh Bipath:** Conceptualization (equal); supervision (equal); writing – review and editing (equal). **Yvette Nkondo Hlophe:** Conceptualization (equal); supervision (equal); writing – review and editing (equal).

## FUNDING INFORMATION

The author(s) disclosed receipt of the following financial support for the research, authorship, and/or publication of this article: The Research Development program of Dr YN Hlophe and Dr JC Serem by the University of Pretoria. School of Medicine Research Committee (RESCOM) Grant awarded to Ms Basson. National Research Foundation (NRF) awarded to Prof R Anguelov.

## CONFLICT OF INTEREST STATEMENT

The author(s) declared no potential conflicts of interest with respect to the research, authorship and/or publication of this article.

## Data Availability

Data sharing not applicable to this article as no datasets were generated or analysed during the current study.
